# Evaluation of the update for screening for retinopathy of prematurity in a tertiary care center in Türkiye with retrospective cohorts

**DOI:** 10.55730/1300-0144.5912

**Published:** 2024-10-20

**Authors:** Emine KAYA GÜNER, Duygu İNCİ BOZBIYIK

**Affiliations:** Department of Ophthalmology, Faculty of Medicine, University of Health Sciences, Tepecik Research and Training Hospital, İzmir, Turkiye

**Keywords:** Guideline update, retinopathy of prematurity, screening, Türkiye cohort

## Abstract

**Background/aim:**

In Türkiye, the recommendations for screening for retinopathy of prematurity (ROP) were updated in 2021. We aimed to present detailed data on the infants included in the screening program according to the new criteria and evaluate whether these changes are of benefit in detecting severe ROP.

**Materials and methods:**

Our hospital’s medical records of infants screened for ROP between July 2019 and July 2021 or between August 2021 and August 2023 were retrospectively examined. Gestational age (GA), birth weight, the sex of the infant, whether there was multiple pregnancy, ROP examination results, the most advanced ROP level and time, and ROP treatment needs and times were recorded. Cohort data from these two time periods before and after the update were compared.

**Results:**

Three hundred and fifty-seven infants screened before the updating of the guidelines and 336 infants screened after the update were included in the analysis. Between August 2021 and August 2023, more cases of ROP were detected (19.3% and 21.4% for the two cohorts, respectively), while a lower rate of treatment was required (3.9% and 2.1% for the cohorts, respectively). One of the infants treated after the update was included in the screening based on the new GA criterion. In both cohorts, no infant needed treatment before 32 weeks of postmenstrual age regardless of agreement with GA at birth.

**Conclusion:**

In this study, one infant in need of treatment who was not included in the screening program according to the previous criteria was identified. The data we obtained support the necessity of increasing the upper limit for GA. Additionally, the 31st week seems safe for the beginning of examinations in extremely premature infants.

## 1. Introduction

Retinopathy of prematurity (ROP) is a physiopathological condition occurring due to abnormal proliferation of retinal vessels in premature infants. Its pathogenesis remains unclear. ROP is one of the leading causes of preventable blindness and visual impairment in childhood [1]. Recent studies suggest that ROP has become the leading cause of pediatric blindness in developing countries [2].

The frequency of ROP varies depending on countries’ development levels and the characteristics of neonatal intensive care units. Therefore, guidelines for ROP screening vary between countries, with no unified international screening criteria available. In published guidelines for different countries, criteria ranging from 30 to 37 weeks for gestational age (GA) and 1500 to 2000 g for birth weight (BW) are used [3–7]. Additionally, regional guidelines need to be constantly evaluated and modified as necessary [8].

Guidelines for ROP screening are based on population-based studies and are subject to change based on current data. Studies conducted in Türkiye have reported that severe ROP can also develop in infants with GA of 33–35 weeks and BW of 1500–2000 g [5]. Therefore, the ROP diagnosis and treatment guidelines created in Türkiye in 2016 were updated in 2021. According to the 2016 recommendations, all infants born with a GA of ≤32 weeks or BW of ≤1500 g and preterm infants born with GA of >32 weeks or BW of >1500 g who were on cardiopulmonary support therapy or “deemed at risk for the development of ROP by the clinician monitoring the infant” should be screened [9]. Moreover, the first ophthalmological examination was performed at the 30th or 31st postmenstrual (PM) week for infants whose GA was less than 27 weeks. After the publication of the Turkish Society of Neonatology and Turkish Ophthalmology Society Retinopathy of Prematurity Updated Guidelines in 2021, the criteria encompassed GA of less than 34 weeks, BW of less than 1700 g, or poor postnatal clinical outcome as determined by the neonatologist [10]. There has been no change in the general recommendation for the infant’s first ophthalmological examination date. However, it is now recommended that the first examination can be performed at the 6th postnatal week without waiting for the 31st PM week in infants with GA of less than 25 weeks [10].

In guideline updates in developed countries, the upper GA and BW limits for inclusion in screening have generally been lowered, whereas in Türkiye, they needed to be increased [5,8,10,11]. In this study, we aimed to examine the rate and severity of ROP development in infants during time intervals in which the old and the new criteria were applied and to evaluate detailed data of the infants included in screening according to the new criteria to determine whether the new criteria are of benefit in detecting severe ROP in a tertiary care center with a retrospective cohort.

## 2. Materials and methods

Approval was received from the Health Sciences University İzmir Tepecik Training and Research Hospital Ethics Committee for this study, which was designed as a retrospective cohort (Decision No: 2023/09-39) in compliance with the principles of the Declaration of Helsinki.

The medical records of all infants who were screened for ROP in the ophthalmology clinic of the University of Health Sciences İzmir Tepecik Training and Research Hospital between July 2019 and July 2021 or between August 2021 and August 2023, who were hospitalized in the neonatal intensive care unit of our hospital or who had been presented as outpatients for screening, were retrospectively examined. The study center screened all infants according to the recommendations of the ROP diagnosis and treatment guidelines established in 2016 before July 2021 [9]. These evaluations were performed at the 4th postnatal week for infants born at ≥27 weeks [9,12]. After the publication of the Turkish Society of Neonatology and Turkish Ophthalmology Society Updated Retinopathy of Prematurity Guidelines in 2021, evaluations were performed in line with the new suggestions [10]. All infants screened for ROP between August 2021 and August 2023 according to the updated criteria and whose results were fully documented were included in the analysis. Infants with missing data or who exited the ROP screening process before its completion were excluded.

The first ROP screening examination for the included infants was performed at least 4 weeks after birth or at the 31st PM week. After July 2021, in line with the new recommendations, infants with GA of <25 weeks were first examined after the 6th postnatal week without waiting for the 31st PM week. The same protocol was applied for all newborns and patients were examined by ophthalmologists with experience in ROP. One hour after instilling mydriatic eye drops (0.5% tropicamide and 1% phenylephrine), binocular indirect ophthalmoscopy was performed with a 28-diopter lens using a sterile eyelid speculum and scleral depressor. The classification of ROP was based on the International Classification of Retinopathy of Prematurity, and indication for treatment was based on the Early Treatment for Retinopathy of Prematurity Study [13,14]. Follow-up was performed according to the recommended follow-up plan until complete retinal vascularization or regression of ROP, or until treatment was required [3,13].

ROP results representing the most advanced stage reached in the course of the disease for each patient were classified as severe ROP [type 1 ROP, aggressive ROP (A-ROP), and ROP requiring treatment], type 2 ROP, and low-grade ROP [10,13]. Low-grade ROP was defined as ROP findings at stages outside the criteria for type 1 ROP, A-ROP, or type 2 ROP. Gestational age (GA), birth weight, the sex of the infant, whether there was multiple pregnancy, ROP examination results, the most advanced ROP level and time, and ROP treatment needs and times were recorded.

Statistical analysis of the obtained data was performed using IBM SPSS Statistics 24.0 (IBM Corp., Armonk, NY, USA). The compliance of the data to normal distribution was examined visually with histograms and probability graphs (PP plot) and analytically with the Kolmogorov–Smirnov test (n > 50). Descriptive data were presented as mean ± standard deviation or median [interquartile range (IQR)] values for numerical variables according to their normality. Normally distributed numerical variables were compared with a t-test or ANOVA, while nonnormally distributed variables were compared with the Mann–Whitney U test or Kruskal–Wallis test. Categorical data were presented as numbers and percentages and were compared using Pearson and chi-square tests. Values of p < 0.05 were considered statistically significant.

## 3. Results

A total of 361 infants underwent ROP screening examinations between July 2019 and July 2021. Three of these patients were excluded from the study because they died before their examinations could be finalized and one patient was excluded because of missing data. The demographic data of the 357 analyzed cases are given in Table 1. Sixty-nine (19.3%) of these infants developed ROP and the stages of these cases are given in Table 2. Treatment was required in 14 cases (3.9%). Times at the onset of maximum ROP and at the first needed treatment are given in Table 3. There was no statistically significant difference in ROP development according to sex or multiple pregnancy (p = 0.589 and p = 0.74, respectively). There was a statistically significant difference in the development of ROP according to GA and BW (p < 0.0001 for both). There were also statistically significant differences in the severity of ROP and the need for treatment according to both GA and BW (p < 0.0001 for all). More cases of ROP and more severe ROP were detected in infants with lower GA and BW, and more frequent treatment was required.

ROP screening examinations were performed for a total of 339 infants between August 2021 and August 2023. Two of these patients died before their examinations could be completed and one patient was excluded from the study due to missing data. The demographic data of the 336 analyzed cases are given in Table 1. Seventy-two (21.4%) of these infants developed ROP and the stages of these cases are given in Table 2. Seven (2.1%) infants required treatment. Times at the onset of maximum ROP and at the first needed treatment are given in Table 3. No statistically significant difference was observed for the development of ROP according to sex or multiple pregnancy (p = 0.467 and p = 0.567, respectively). There was a statistically significant difference in the development of ROP according to both GA and BW (p < 0.0001 for both). There was also a statistically significant difference in the severity of ROP according to GA and BW (p = 0.010 and p = 0.013, respectively) and a statistically significant difference in the need for treatment according to GA and BW (p < 0.0001 and p = 0.001, respectively). In this second cohort, more cases of ROP and more severe ROP were detected in infants with lower GA and BW, and more frequent treatment was required.

When the data of the infants examined in the scope of the screening criteria of the updated 2021 Turkish Retinopathy of Prematurity Guidelines were analyzed in detail, it was found that 150 patients (51.3% male and 48.7% female) were screened between August 2021 and August 2023 because they were born at 32–34 weeks, and 31 patients (54.8% male and 45.2% female) were screened because they were born at 1500–1700 g. Twenty-five patients born at 1500–1700 g were already included in the screening program because their GA was less than 34 weeks. When the data of the infants screened according to the GA criterion were examined in detail, the mean BW was 2171.38 ± 431.261 g and the mean GA was 33.37 ± 0.53 weeks. ROP developed in 16 (10.7%) cases, and 1 (0.7%) of these infants had severe ROP that required treatment. The treated infant was born at 33 weeks weighing 1790 g and had stage 3 plus ROP examination findings in zone 2 (anterior). The other 15 (10%) patients developed low-grade ROP. When the data of the infants included in the screening based on the weight criterion were examined in detail, the mean GA was 32.25 ± 2.23 weeks and the mean BW was 1626.35 ± 57.13 g. ROP developed in 6 of these patients (19.4%) and all 6 of these cases were low-grade ROP. None of these patients required treatment.

## 4. Discussion

Improvements in neonatal care and the resulting increase in survival rates have led to an increased prevalence of ROP in developing countries. Early diagnosis and treatment of this disease are critical in preventing blindness [1,3]. For this reason, effective screening for ROP is essential. Screening examinations are stressful for premature infants and are also costly as they require an experienced team and appropriate equipment. For these reasons, the upper GA and BW limits for inclusion in screening tend to be reduced in guideline updates in developed countries to avoid unnecessary screening examinations [8,11]. On the contrary, in developing countries such as Türkiye and Mexico, it has been necessary to increase the upper limits for inclusion in screening [10,15].

Most epidemiological studies on ROP are limited to local regions or single countries [16]. There are many studies from different countries reporting ROP rates between 15.9% and 45.2% [5,7,8,17–22]. Since these rates were presented as percentages, they were affected by the population size included in each study and, naturally, the inclusion criteria. In a prospective multicenter study from Türkiye, the incidence of ROP was 27% and the rate of needing treatment was 6.7% [5]. In the present study, when data obtained before and after the updating of the guidelines were evaluated, a higher incidence of ROP (19.3% and 21.4% for the two cohorts, respectively) and a lower need for treatment (3.9% and 2.1% for the cohorts, respectively) were observed after the update. When the cohorts before and after the updating of the guidelines were compared, GA at birth (32.19 and 32.63 weeks, respectively) was almost the same, while BW (1819.86 and 1994.55 g, respectively) was higher in the cohort formed after the update. The incidence rates of ROP at any stage and ROP requiring treatment were lower in each of these cohorts than in the aforementioned multicenter study [5]. The screening of more mature infants due to higher upper limits may have caused more mild cases of ROP to be diagnosed. Additionally, although both of these studies were conducted in Türkiye, the present study offers single-center data from a large urban tertiary hospital with good standardization of neonatal care. Thus, the differences may have resulted from different practices in neonatal intensive care or may reflect differences in sociodemographic characteristics since the previous study was a multicenter study. Different criteria may be used for screening in different regions, even in the same country.

In this study, both before and after the updating of the guidelines, infants with lower GA and BW had more ROP and more severe ROP and needed treatment more frequently, similar to the literature [7,23,24]. Similar to the CRYO-ROP study and a New York cohort study, there was no difference in ROP development according to sex before or after the update [7,23,25]. However, some studies in the literature report that being male is a significant risk factor for ROP [26–28]. Unlike the CRYO-ROP study [23], we observed no difference in ROP development according to multiple pregnancies in either cohort. Although some studies have concluded that multiple pregnancies are significantly associated with ROP, others have reported no difference, as in our study [29–32]. These differences may be due to a lack of adjustment for maternal risk factors, perinatal treatment, and infant risk factors.

After the updating of the guidelines, 7 of 336 screened infants developed severe ROP and required treatment. One of those 7 infants was born between 32 and 34 weeks; therefore, this infant was included in the screening based on the new GA criterion. In the TR-ROP study, in which the data of 6115 infants across Türkiye were prospectively examined, severe ROP developed in 5 infants with BW of >1500 g and GA of >32 weeks [5]. In another study performed in Türkiye, among 1784 infants with BW of >1500 g, the rate of development of ROP of any stage was 14.1%, while the rate of development of severe ROP was 0.6% [33]. In our study, the GA of the two infants who needed treatment was over 32 weeks (both with a GA of 33 weeks). Although our rate of development of any stage of ROP or severe ROP after the updating of the guidelines was lower compared to the aforementioned studies, one infant with a GA of over 32 weeks needed treatment in our study. A study from, India, another middle-income country, reported a 15.3% incidence rate among infants weighing >1500 g who were treated with cryotherapy for threshold ROP [34]. In a study from Brazil, Freitas et al. reported that only 8 out of 602 patients with a GA of >32 weeks or BW of >1500 g developed ROP, and all of those cases were stage 1 [35]. There are no previous studies evaluating ROP in infants with GA of 32–34 weeks and BW of 1500–1700 g. However, when data on more mature infants in developing countries are examined, it is seen that the subjective criterion of neonatologists referring infants who are considered risky above the upper limits of GA and BW is still very important. It can be argued that this loss of specificity in screening creates examination-related stress in infants and constitutes a financial burden, especially for developing countries. However, because missing severe cases of ROP has devastating consequences, overscreening is considered acceptable.

In this study, the time to reach the maximum ROP level and the timing of treatment were very similar between the cohorts. The updated guidelines recommend that screening examinations can start at 6 weeks for infants born before 25 weeks. The EXPRESS study, a national study of extremely preterm infants born before 27 weeks in Sweden, recommended delaying the first examination in these immature infants until the age of 31 PM weeks [36]. In both cohorts of our study, no infant needed treatment before 31 weeks of PM age, and no infant needed treatment before 32 weeks of PM age regardless of agreement with GA at birth. In another cohort study from Sweden, the development of stage 3 ROP occurred at an average of 36.7 (range: 31.1–52.6) weeks and the need for treatment arose at an average of 37.4 (range 32.1–51.4) weeks [8]. Similarly, in another study from Hong Kong, the mean duration to treatment for ROP was reported as 40 weeks of GA (range: 35–48 weeks) [37]. The 31st week seems safe for the beginning of these examinations in extremely premature infants.

This study has several limitations including its retrospective design and single-center nature. However, this study is the first in the literature to evaluate the infants included in ROP screening after the updating of the guidelines in Türkiye. Therefore, developing further screening guidelines in this country regarding the infants to be included in the scope of ROP screening may be valuable.

In conclusion, although increasing the upper limit of GA for inclusion in ROP screening leads to an increase in workload and costs, it is vital for public health that infants in need of treatment be included in screening programs according to the criteria. Although the data we obtained are based on a single center, they support the necessity of increasing the upper GA limit.

## Figures and Tables

**Table 1:** Characteristics of the study population.

Characteristic	2019–2021 (n = 357)	2021–2023 (n = 336)

Male/Female	181–176	188–148

Birth weight, g		

Mean *(SD)*	1819.86 (536.48)	1994.55 (567.49)

Median (1^st^ quartile–3^rd^ quartile) [*range*]	1800 (1438–2192.50) [450–3670]	2073 (1600–2378.75) [600–3500]

Gestational age, wk		

Mean (SD)	32.19 (2.33)	32.63 (2.38)

Median (1^st^ quartile–3^rd^ quartile) [range]	33.00 (31.00–34.00) [24–39]	33.14 (31.28–34.00) [24.57–39.14]

Number of birth, n		

singleton	264	276
twin	86	58
triplets	7	2

Abbreviation: ROP, retinopathy of prematurity.

**Table 2:** Stage of ROP in the cohorts from 2019–2021 and 2021–2023, respectively.

	All Patients n	No ROP n (%)	Low-grade ROP n (%)	Type 2 ROP n (%)	Severe ROP n (%)

2019–2021	357	288 (80.7%)	54 (15.1%)	1 (0.3%)	14 (3.9%)
2021–2023	336	264 (78.6%)	63 (18.8%)	2 (0.6%)	7 (2.1%)

**Table 3:** Time at onset of maximum ROP stage and at first treatment in the cohorts from 2019–2021 and 2021–2023, respectively.

	2019–2021 (n = 357)	2021–2023 (n = 336)
Diagnosis max-age, wk		
Mean (*SD*)	38.76 (3.50)	38.70 (3.50)
Median (1^st^ quartile-3^rd^ quartile) [range]	38.50 (36.20–40.96) [32–49.71]	37.64 (35.07–40.00) [31–50.28]
Need for treatment, wk		
Mean (*SD*)	37.50 (3.50)	36.81 (2.53)
Median (1^st^ quartile-3^rd^ quartile) [range]	37.14 (35.46–39.50) [32.70–46.20]	37.85 (34.00–38.85) [32.57–39.14]
